# Needle Tip Visibility in 3D Ultrasound Images

**DOI:** 10.1007/s00270-017-1798-7

**Published:** 2017-09-19

**Authors:** Muhammad Arif, Adriaan Moelker, Theo van Walsum

**Affiliations:** 1 Department of Medical Informatics, Erasmus MC, University Medical Center Rotterdam, Wytemaweg 80, Room Na 2506 Erasmus MC, 3015 CN Rotterdam, The Netherlands; 2 Department of Radiology and Nuclear Medicine, Erasmus MC, University Medical Center Rotterdam, Rotterdam, The Netherlands

**Keywords:** Needle tip, Ultrasound, Visibility, Contrast-to-noise-ratio (CNR), Image guidance

## Abstract

**Aim:**

Needle visibility is crucial for effective and safe ultrasound-guided interventional procedures. Several studies have investigated needle visibility in 2D ultrasound imaging, but less information is available for 3D ultrasound imaging, a modality that has great potential for image guidance interventions. We performed a prospective study, to quantitatively compare the echogenicity of various commercially available needles in 3D ultrasound images used in clinical practice under freehand needle introduction.

**Materials and Methods:**

A set of seven needles, containing biopsy needles, a TIPS needle, an ablation needle and a puncture needle, were included in the study. A liver-mimicking phantom and cow liver were punctured by each needle. 3D sweeps and real-time 3D data were acquired at three different angles (20°, 55° and 90°). Needle visibility was quantified by calculating contrast-to-noise ratio.

**Results:**

In the liver-mimicking phantom, all needles showed better visibility than in the cow liver. At large angles, contrast-to-noise ratio and needle visibility were almost similar in both cases, but at lower angles differences in visibility were observed with different types of needles.

**Conclusion:**

The contrast-to-noise ratio increased with the increase in angle of insonation. The difference in visibility of different needles is more pronounced at 20° angle. The echogenic properties of inhomogeneous cow liver tissues make the needles visibility worse as compared to a homogenous phantom. The needle visibility becomes worse in 3D real-time data as compared to 3D ultrasound sweeps.

## Introduction

Needle puncture is an important part of interventional radiology procedures. Such procedures are generally image guided, where the images are used to visualize the anatomy (target) and the needle. Fluoroscopy and ultrasound (US) are common imaging modalities used for image guidance in minimally invasive procedure, and both allow real-time imaging. Whereas fluoroscopy uses ionizing radiation and expensive C-arms, US is a safe and relatively cheap modality and is most frequently used for needle interventions.

Conventionally, radiologists use 2D US to visualize the needle during insertion. There are mainly two approaches for needle insertion: guided and freehand [1]. In the guided needle approach, a detachable guide is attached onto the 2D US probe and guidance lines on the US screen show the expected path of the needle through the tissue. In freehand approach, needles are inserted through the tissue without the use of a guide. The freehand technique is more challenging than the guided approach but it provides greater flexibility in choosing the introduction angle of the needle. The visibility of the needle using US guidance is of hallmark importance and can become limited in tissues with increased echogenicity, with depth or in presence of air or bone [2]. A small angle of ultrasound beam with the needles and needles with small diameter (gauge) also make visualization difficult [2, 3]. A good needle visibility can help in avoiding unintended tissue damage. Several techniques have been reported in the literature to improve the visibility of needles in 2D ultrasound image guidance. Kawai et al. [4] used a commercially available vascular needle (20 gauge) and created four modified versions: a re-cut needle, a dimple needle, a file-like rough surface needle and a needle with four sides holes. They evaluated the 2D US images, and the visibility was graded from invisible to excellent by interventional radiologists. Maecken et al. [5] used 12 different needles and rated the visibility in 2D US images using a categorical visibility score. Nichols et al. [3] also compared the echogenicity of several types of needles at different angles of insonation in 2D ultrasound images.

Real-time 3D US is a relatively novel imaging modality that permits to visualize tissue in 3D. In a typical 3D US data acquisition, the sonographer scans the body with a single sweep with a volumetric 3D transducer. Each 3D US scan consists of multiple parallel 2D US planes, which allows examination of anatomical structures in any plane. Traditionally, 3D US used in many applications such as abdominal imaging to measure liver masses and in gynaecology to examine human foetus. 3D US allows the measurement of organs and lesions volumes. It also has a great potential for guidance in complex interventional procedure [6]. 3D ultrasound imaging also permits freehand needle introduction, but image guidance may become more difficult. Furthermore, 3D US may compromise needle tip visibility and would benefit from needle tip position detection and tracking. Therefore in this study, we investigated the needle tip visibility in 3D US imaging. We performed in vitro and in vivo experiments with different needles commonly used in interventional radiology procedures and quantitatively assessed needle tip visibility in 3D ultrasound images using image processing.

## Materials and Methods

### Needles

**Fig. 1:**
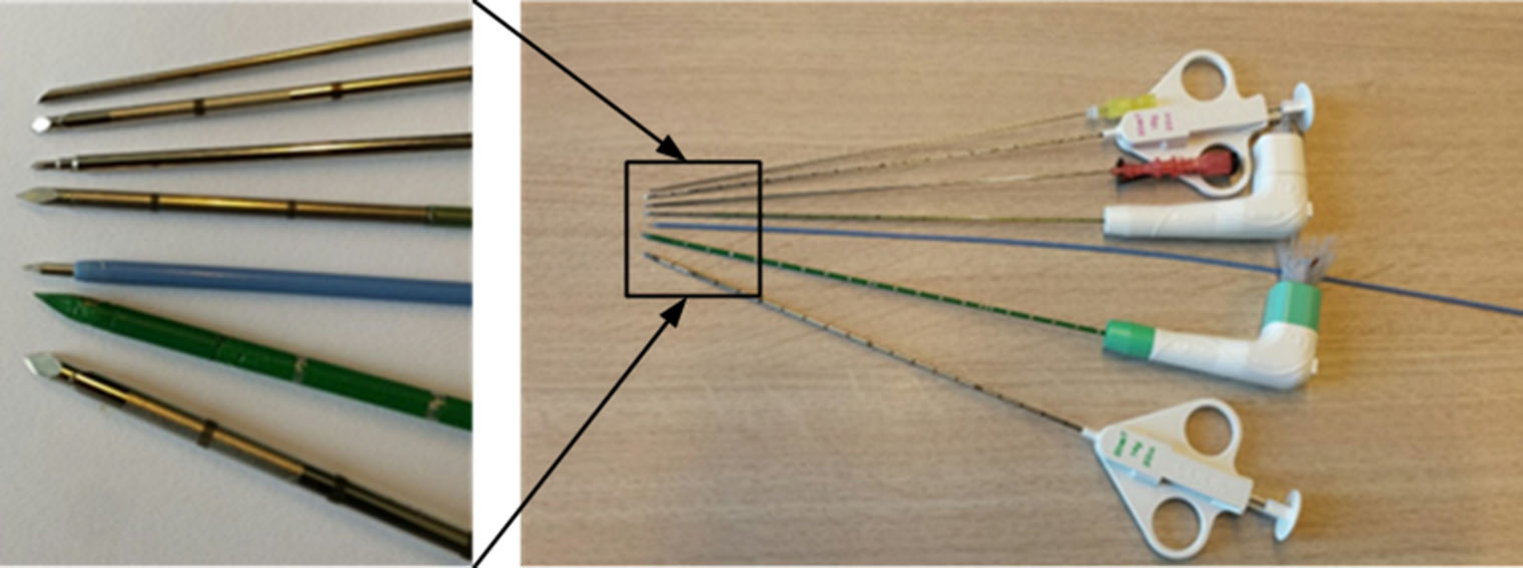
Commercial needles used in experiments

Seven needles frequently used in interventional procedure with different length (mm) and gauge were used in the experiment (see Fig. 1). These seven needles consist of three biopsy needles: Quick-Core (14G, 18G), Chiba (20G), one TIPS needle, two ablation needles and a puncture needle. More detail of types and characteristics of each needle are provided in Table 1. The biopsy needles: Quick-Core (14G, 18G) and Chiba (20G) contained an echogenic tip, whereas the remaining needles were without any echogenic modification.

**Table 1:** Characteristics of the examined needles

Number	Manufacturer	Name of needle	Gauge	Length (cm)	Diameter (mm)	Tip type
1	Cook	Chiba (biopsy)	20	20	0.81	Echo
2	Cook	Quick-Core (biopsy)	18	20	1.02	Echo
3	Angiomed	Initial puncture needle	17.5	20	1.08	Standard
4	HS	RFA (ablation)	17	20	1.15	Standard
5	Cook	Colapinto (TIPS)	16	505	1.29	Standard
6	HS	MW (ablation)	14	20	1.63	Standard
7	Cook	Quick-Core (biopsy)	14	20	1.63	Echo

Manufacturer = Cook Medical, Bjaeverskov Denmark. HS Hospital Services S.P.A, Aprilia Italy

### Liver-Mimicking Phantom

**Fig. 2:**
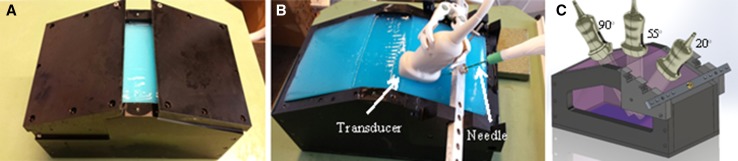
**A** mould used to construct liver-mimicking phantom from PVA. **B** Experimental setup, where MW Ablation needle is inserted into the phantom and US data were acquired at 20° angle. **C** Diagram explaining the US data was acquired at three different angles (20°, 55° and 90°)

A soft tissue (liver)-mimicking phantom was made from a 5% aqueous solution of polyvinyl alcohol (PVA). Silica gel particles (1%) were added in the solution to act as US scatters. PVA solution was heated in water for 30 min and poured in a mould (30 cm in length, 15 cm in width and 16 cm in depth). The mould was first kept at room temperature (24 °C), and some free space at the top of the mould was left to allow air bubbles to escape from the solution (see Fig. 2A). After 6 h of rest, air bubbles disappeared and the mould was stored in a freezer at −20°C. After 14 h in the freezer, the mould was kept at room temperature again for 8–10 h. This procedure constituted one freeze–thaw cycle. The liver model was freeze–thawed two times. The stiffness of the model increased with the number of freeze–thaw cycles [7, 8].

### Cow Livers

Three fresh cow livers (1.6, 1, 1 kg) were used in the experiments. Some parts of the liver were removed to make a suitable shape for the experiment. Each liver was submerged in a water tank during the experimental procedure. The water in the tank acted as US coupling medium.

### Experiments

#### Liver-Mimicking Phantom

Each needle was inserted into the phantom at approximately 10 cm depth by an experienced US researcher (see Fig. 2B). For each needle, we acquired a 3D ultrasound sweep and real-time 3D data at three different angles (20°, 55° and 90°) (see Fig. 2C). The wide range of relevant angles was chosen to see the effect of insonation angle on needle visibility. We obtained 3 US sweeps for each needle saved locally on the US machine, one sweep for each of three angles, leading to 21 US sweeps per experiment. Images were acquired using an ultrasound system (Philips, iU22, the Netherlands) equipped with an X6-1 3D transducer. Recorded 3D sweeps were transferred form the ultrasound machine to a personal computer (PC) for post-processing. The real-time 3D data were directly saved on the PC using an Ethernet connection between the US system and the PC. Needles were scanned axially with the focal zone (focus) of ultrasound beam at the tip of needle. The gain of ultrasound system was adjusted and kept constant for each measurement to acquire good quality images.

#### Cow Liver

**Fig. 3:**
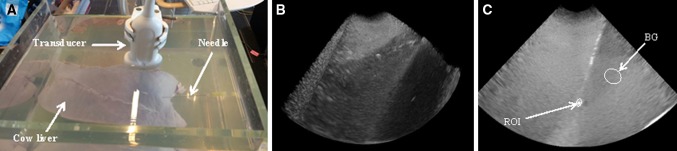
**A** Experimental setup, where a cow liver is submerged in a water tank and US data were acquired at 55° angle. **B** A 3D sweep (volume) acquired using 3D transducer. **C** A 2D slice of 3D US sweep containing the tip of a needle and a region of interest around the needle tip. *FG* Foreground, *BG* background

A cow liver was submerged in a water tank and punctured by each needle to acquire a 3D sweep and real-time data at three different angles (20°, 55° and 90°) as shown in Fig. 3A. Unlike the liver phantom, the size of cow liver was small and water was needed to act as coupling medium. The US data were acquired and processed similar to phantom experiments. US system settings (frequency, gain, depth and focus.) were also set similar as for liver phantom experiment.

### Image Processing/Analysis

The 3D US sweep of each needle was analysed using an in-house application that was built with MeVisLab (MeVis Medical Solution AG, Bremen, Germany). From each sweep shown in Fig. 3B, the 2D slice containing the tip of needle was selected and a region of interest along the needle length and around the needle tip (foreground, FG) and background (BG) were chosen (see Fig. 3C). From these areas, mean pixel intensity values ($\overline{\text{FG}}$ and $\overline{\text{BG}}$) and standard deviation (BG only) were calculated. To quantify needle visibility, we calculated the contrast-to-noise ratio (CNR) for each needle using the following relation.

$${\text{CNR}} = \frac{{\overline{\text{FG}} { - }\overline{\text{BG}} }}{{{\text{std}}\left( {\text{BG}} \right)}}$$

The ellipsoidal ROI had 10 mm (22 pixels) longest and 5 mm (11 pixels) shortest diameter, and using the MeVisLab software the size was kept fixed for all needle images to avoid the influence of ROI size on the mean pixel intensity and consequently to CNR values. The size of ROI was chosen such that it covered the needle tip area for all types of needles used in the experiments.

## Results

### Liver-Mimicking Phantom

**Fig. 4:**
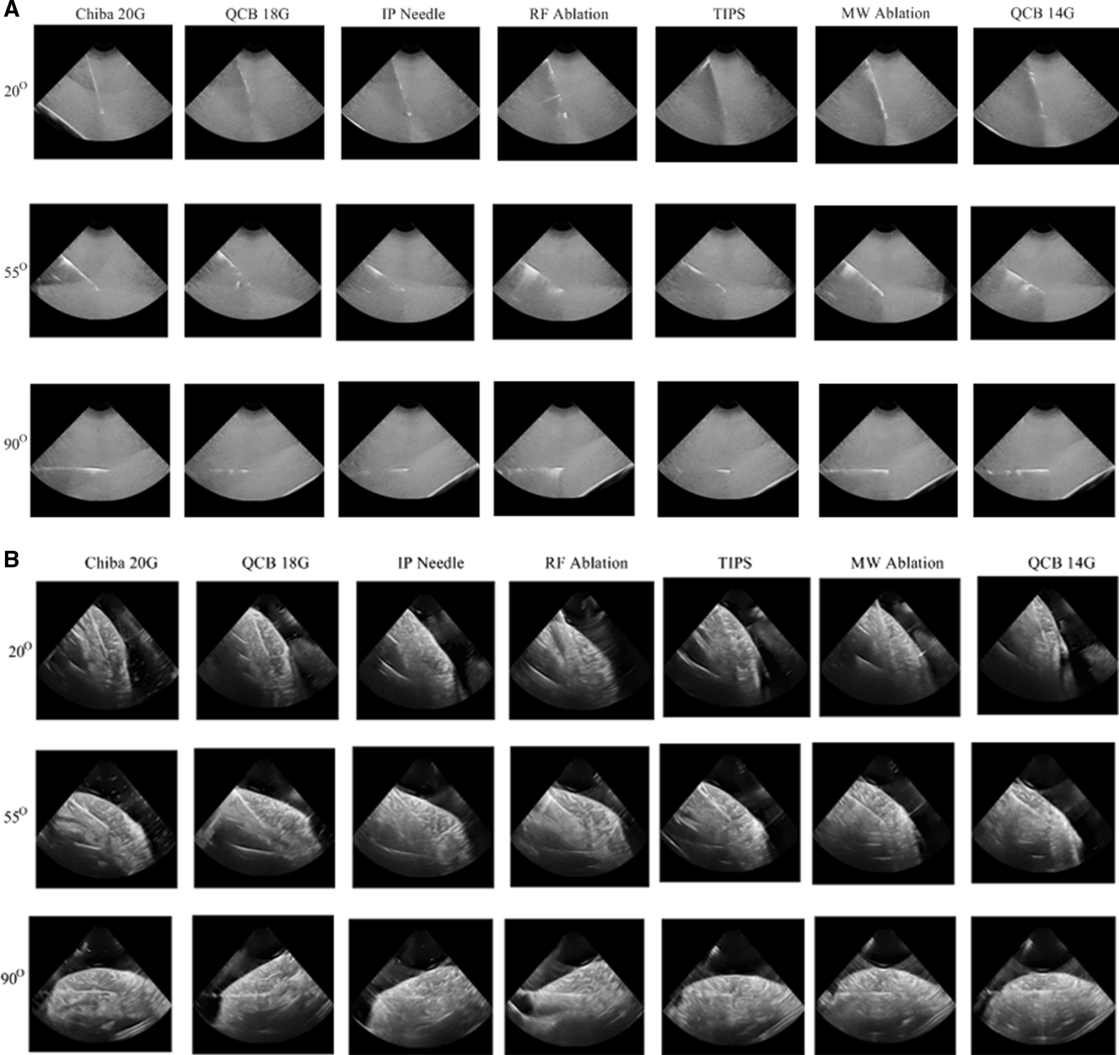
**A** 2D slices from 3D US sweep acquired during scanning of all needles at three different angles (20°, 55° and 90°) in phantom. **B** 2D slices from 3D US sweep acquired during scanning of all needles at three different angles (20°, 55° and 90°) in cow liver

In Fig. 4A, all needles at three different angles in the liver phantom are shown; the needle types are placed in an order of increasing diameter (see also Table 1). There was a small difference between the needles visibility at 90° angles. This shows that large angles are best to have good needle visualization. However, as the angle of insonation decreased, the visibility was also decreased.

**Fig. 5:**
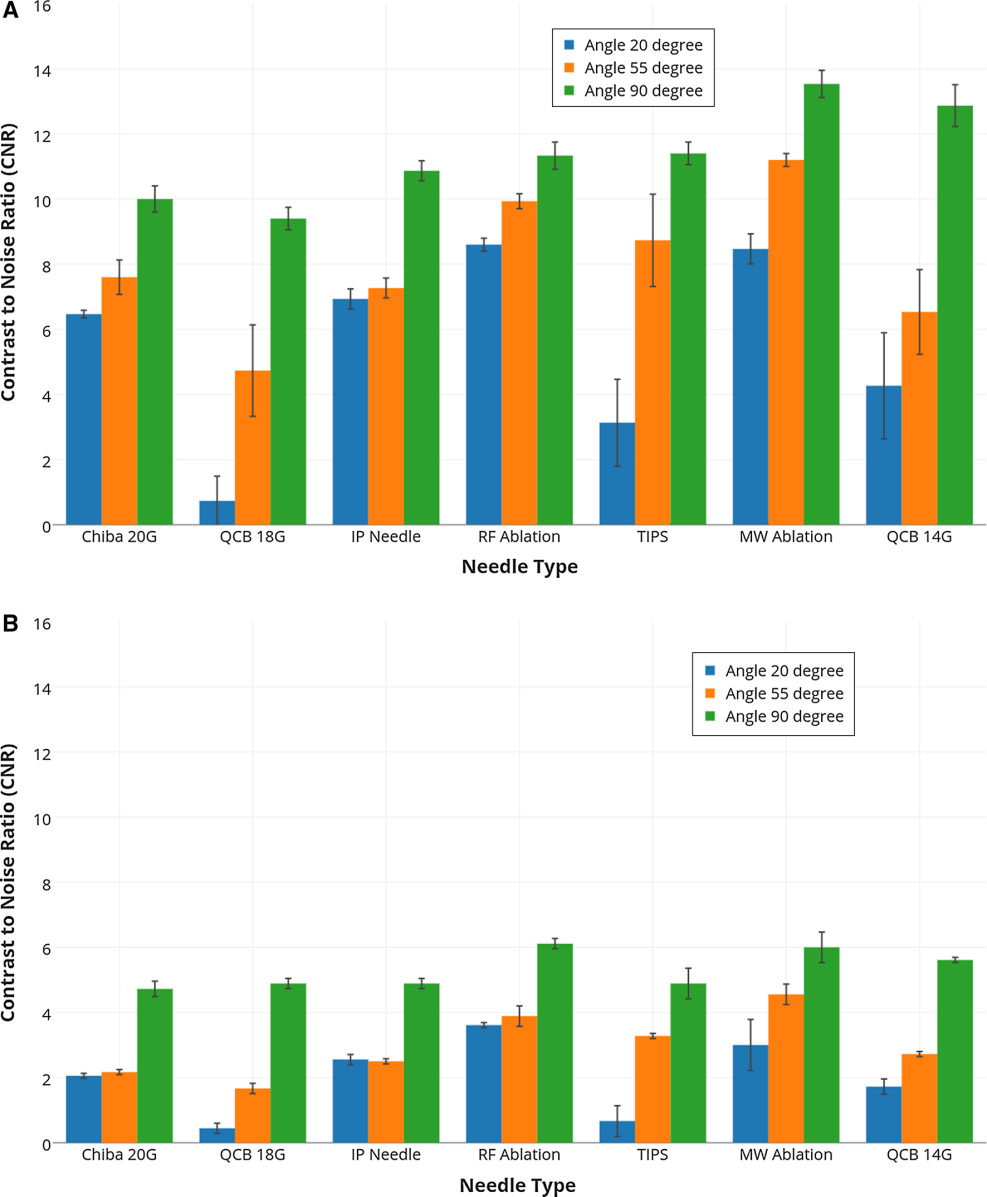
Contrast-to-noise ratio (CNR) for all needles in the phantom at three different angles (20°, 55° and 90°) calculated from **A** 3D US sweeps and **B** real-time 3D data. The experiments were done three times, and the *error bars* represent the standard deviation

The CNR value at large angles also shows a small difference between needles visibility. This shows that at large angles all needles have almost similar echogenicity regardless of their types and size (gauge). However, the difference in visibility becomes apparent at small 20° angle as shown in Fig. 5A.

The real-time 3D data show a similar pattern like 3D US sweeps; however, the CNR values decrease overall due to lower image resolution (see Fig. 5B). The experiments were done three times, and the error bars in Fig. 5 represent the associated standard deviations.

### Cow Liver

At 90° and 55° angle, all needles have almost similar echogenic visibility as shown in Fig. 4B. However at 20° angle, biopsy needles and the TIPS needle have poor echogenic needle tip visibility. Needle echogenicity decreased with the decrease in angle of insonation, but the magnitude of this decrease is different for each needle type. At 20° angle, the difference in echogenicity for all needles is more visible.

**Fig. 6:**
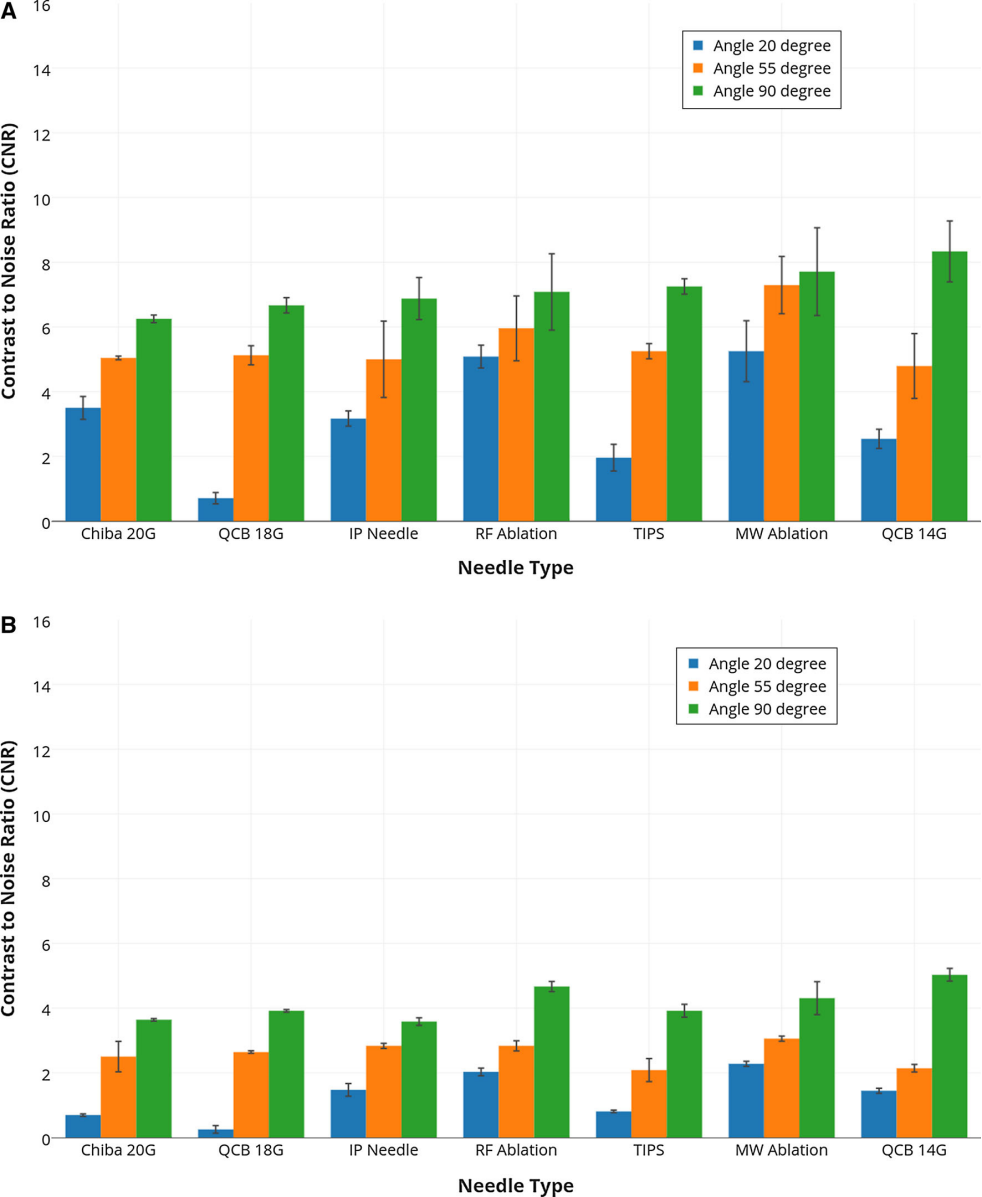
Contrast-to-noise ratio (CNR) for all needles in cow liver at three different angles (20°, 55° and 90°) calculated from **A** 3D US sweeps **B** real-time 3D data. The experiments were done three times and the *error bars* represent the standard deviations

In Fig. 6, we can also see that the CNR values at 20° angle are significantly lower for most of needles than the 55° and 90° angle. The experiments were done three times, and the error bars represent the associated standard deviations.

## Discussion

Several methods have been proposed to enhanced needle tip visibility and improved image guidance in 2D US imaging [2, 9–11]; however, no data are available for 3D ultrasound imaging, a modality that has great potential for image guidance interventions [6].

In the present study, we investigated the visibility of different commercially available needles in 3D ultrasound imaging. The US data were acquired in the form of 3D sweep (one 3D volume) and real-time 3D data (multiple 3D volumes). The images were stored and analysed on a PC. In the study, all needles showed good echogenicity at large angles of insonation. With the decrease in angle between the needle and US beam, the visibility decreased and the difference between needles echogenicity became substantial. Nicholas et al. [3] used a linear 2D transducer to examine the needle visibility in a phantom at different angles. They also found that the echogenicity decreases with the angle, and at large angles all needles have similar echogenic levels. Similar results are also presented in some other studies [5, 9, 12].

**Fig. 7:**
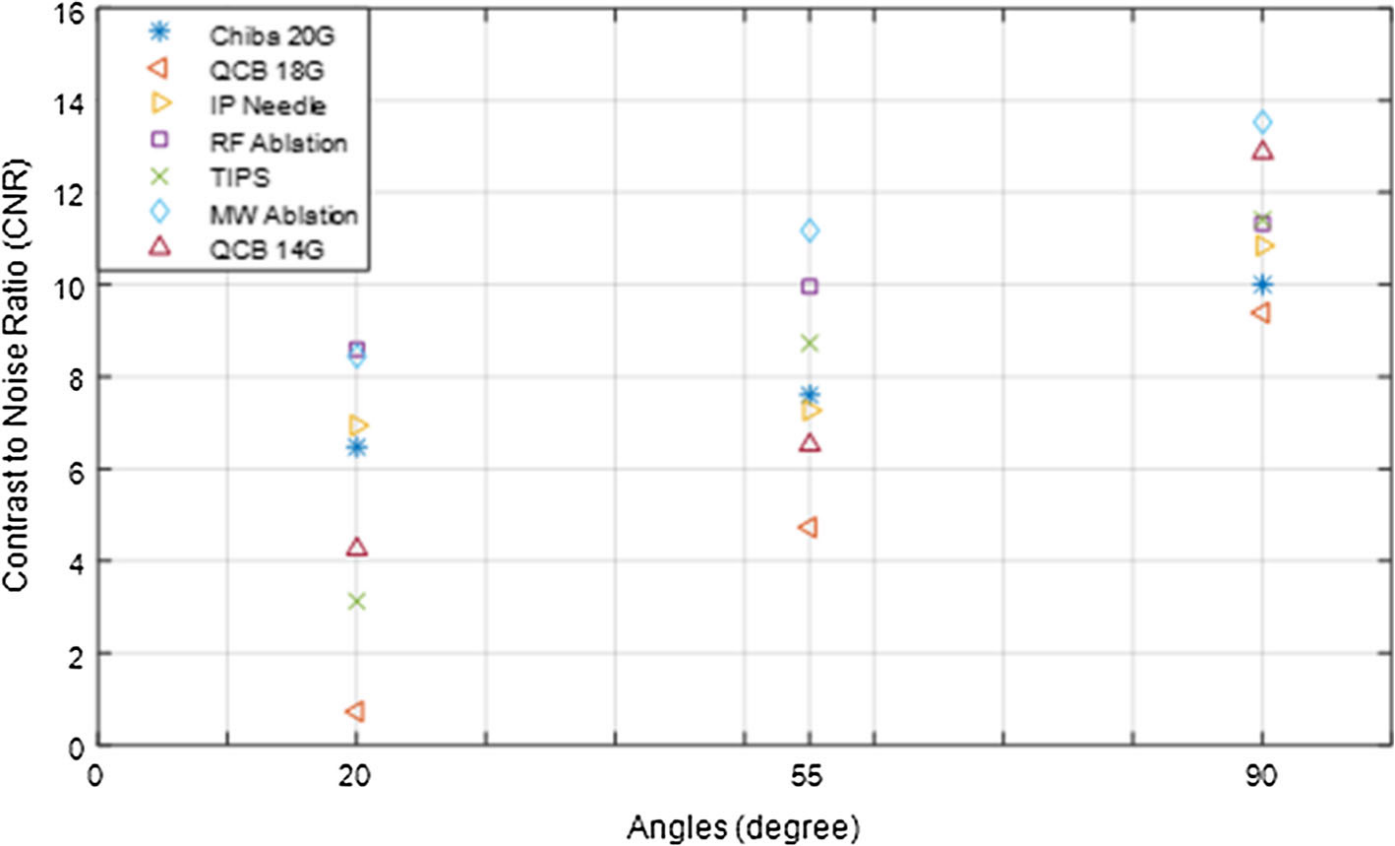
Contrast-to-noise ratio (CNR) values of all needles plotted as a function of three angles

We used different types (with and without echo tip) and sizes (diameter) of needle for our experiments (see Table 1). We plotted the CNR values of all needles as a function of angles and showed that the ablation needle has better visibility (see Fig. 7). We also plotted the CNR as a function of diameter and calculated the R-square values. The results showed that at small angles (20°, 55°) with the increase in diameter of the needle the CNR values and echogenicity do not increase. The *R* ^2^ values were 0.016 and 0.149, respectively, demonstrating no relationship between echogenicity and diameter. However, for 90° angle the *R* ^2^ value was 0.866, which suggests that at higher degree angle echogenicity increases with diameter. Such as for Quick-Core biopsy needle 14G the CNR values are smaller for RF ablation needle at lower angles (20° and 55°). Hopkins et al. [9] performed 2D US imaging on a phantom and showed that at more depth (3 cm) the echo tip and high-gauge needle do not have any advantage over standard needle.

In previous studies, the visibility of needles was scored based on grading of observers [4, 5]. Visual perception of the needle tips on US images depends on multiple factors, such as tissue echogenic properties, formation of artifacts, equipment quality. These factors make the needle visibility comparison difficult. Some used quantitative approach to compare the visibility of needles [2, 3], but an accepted standard method for measuring the echogenicity has not yet been adopted. We used contrast-to-noise ratio (CNR) to quantify needle visibility in 3D US imaging. In image processing, CNR is used to measure image quality based on the contrast in the image where contrast is the difference between signals in two regions. To quantify needle visibility in the region of interest (tip of needle), the difference between needle signal and background signal could provide a good needle echogenic level as compared to tissues.

The ablation needles showed good visibility in US images and CNR values for all angles. Figures 5 and 7 show that CNR values are larger in 3D sweep than the real-time 3D volumes. The reason for this difference is that the 3D sweeps were acquired at higher frame rate (26 Hz for the 2D images, i.e. >1 s per 3D volume) as compared to the real-time 3D data (6 Hz for 3D images). The higher 3D frame rate causes lower image quality and leads to low CNR values. However, similar pattern of CNR values at different insonation angles was obtained in real-time 3D images.

A standard phantom for needle visibility experiments has not been reported in literature, and various materials including agar, gelatine, sponge in water tank and PVA were used in previous experiments. In this study, we used PVA to make a liver-mimicking phantom and examined needles visibility. In our liver phantom, we used 1% silica gel particles to mimic reflection properties of human tissue. However, the variation in the concentration of silica gel could affect the visibility and CNR values. A higher usage of particle concentration could limit the visibility of needle. Also the liver-mimicking phantom lacked the structure features of cow liver; therefore, the experiments were repeated in fresh cow liver for a comparison. The results show that the CNR values are lower in cow liver than the phantom (see Figs. 5; 6). This decrease was due to large background noise caused by the inhomogeneous structure of cow liver. However, the results of phantom and cow liver both showed similar pattern.

The present study shows the findings of an in vitro and ex vivo experiments. In vivo data acquisition and analysis needs to be done in future to compare the results and to make it applicable in clinical practices.

## Conclusion

All examined needles showed good echogenicity at large angles between US beam and needle tip using 3D data acquisition. However, with the decreases in angle all needles become less visible, particularly in cow liver tissue. The needle diameter has no relation with echogenicity at small angles. Ablation needles (microware and radiofrequency) seem to provide good visibility at all angles compared to other types of needles.
